# Clinical Diagnosis of Slipped Capital Femoral Epiphysis in a Child With Negative Radiological Findings: A Case Report

**DOI:** 10.7759/cureus.33396

**Published:** 2023-01-05

**Authors:** Hamad S Alhassan, Youssef B Almushait, Abdullah Y Almarshad, Atheer Alghamdi, Thamer S Alhussainan

**Affiliations:** 1 College of Medicine, Alfaisal University, Riyadh, SAU; 2 Orthopaedics, King Faisal Specialist Hospital and Research Centre, Riyadh, SAU

**Keywords:** negative magnetic resonance imaging, negative xray, delayed diagnosis, clinical diagnosis, slipped capital femoral epiphysis

## Abstract

Slipped capital femoral epiphyses (SCFE) is considered to be a very common disorder among adolescent age group. Multiple risk factors have been reported such as obesity, endocrine disorders, vitamin D deficiency, and panhypopituitarism. The diagnosis of SCFE is important especially in its early stages as this would prevent complications and delay in surgical intervention. The diagnosis is mainly done by radiological imaging and clinical evaluation. However, clinical evaluation is often overlooked. Herein, we present a case of a seven-year-old with SCFE that was diagnosed late due to negative radiological imaging and received late surgical intervention. Therefore, it is recommended that orthopedic surgeons use their clinical sense and examination skills to diagnose SCFE promptly, in order to maintain a short follow-up window to prevent any delay in surgical management and to observe for any progression, even if the radiological findings are normal.

## Introduction

Slipped capital femoral epiphysis (SCFE) is characterized by the displacement of the femoral neck in relation to the femoral head through the epiphyseal plate during rapid growth [1]. It is the most common hip disorder among adolescents [1]. However, SCFE can affect children less than 10 years old [2]. SCFE is classified depending on stability (stable or unstable) [3] and is associated with obesity and endocrine disorders, such as growth hormone deficiency, vitamin D deficiency, and panhypopituitarism [4].

Clinical presentation commonly involves hip, thigh, and knee pain [4], and primary treatment aims to prevent progression of the slip by surgical stabilization: in-situ fixation of the epiphysis with pins or a screw [5]. Early diagnosis of SCFE is necessary to prevent complications like avascular necrosis (AVN), chondrolysis, and deformity [6-7]. SCFE diagnoses are done mainly by proper clinical evaluation and imaging [8]; the use of MRI helps in early diagnosis when plain film radiography results are normal [9]. However, in most cases, clinical examinations are overlooked when attempting to diagnose SCFE. We reported the case of a seven-year-old girl with a high clinical suspicion of SCFE and negative imaging findings, including MRI; this resulted in delayed diagnosis and surgical intervention.

## Case presentation

At the age of three, the patient was diagnosed with hemophagocytic lymphohistiocytosis (HLH), failure to thrive, recurrent infections, skeletal dysplasia, chronic diarrhea, anemia, and neutropenia. As per the HLH protocol, she was treated with IV immunoglobulin and dexamethasone. A bone marrow transplant from an unrelated donor was performed when she was five years old. However, at that time, her marrow transplant was complicated by engraftment syndrome, mild pericardial effusion, and acute kidney injury. At the age of seven, during endocrinology follow-up for her short stature, she complained of limping and two months of bilateral hip pain. The endocrinologist advised to stop growth hormone therapy and referred her to orthopedic surgery for her painful limp.

**Figure 1:**
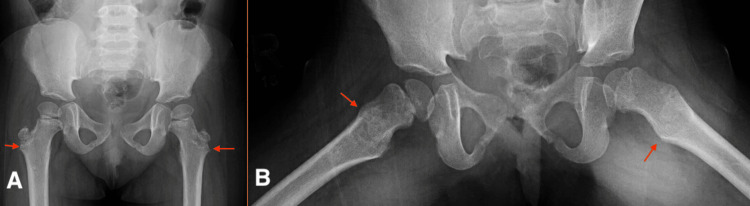
AP pelvis (A) and frog legs lateral (B) X-ray. Bilateral iliac crest as well as femoral metaphyseal irregularity (red arrows), likely related to the known renal disease. Visualized bones and joint spaces are unremarkable. No fracture or dislocation. No gross soft tissue abnormality. AP, anteroposterior

Upon orthopedic surgical evaluation, a mild limp was noted, alongside intermittent bilateral hip pain without a history of recent trauma, infection, or familial musculoskeletal disorders. On physical examination, she was walking with an out-toeing gait, and bilateral hip flexion was limited to 100 degrees, internal hip rotation limited to less than 10 degrees, external rotation of the hip increased to more than 90 degrees, hip obligatory external rotation during passive hip flexion, and femoral anteversion was neutral. However, her blood test results were unremarkable. Given the patient’s age and lack of radiological findings on her anteroposterior (AP) pelvis and frog legs lateral X-ray report (Figure 1A,B), SCFE was deemed unlikely. The X-ray was reviewed by a senior pediatric orthopedic surgeon and musculoskeletal radiologist, who were both unable to uncover any radiological findings suggestive of SCFE. Other differential diagnoses such as septic arthritis, toxic synovitis, and developmental dysplasia of the hip were ruled out. However, given a high clinical suspicion of SCFE, an MRI was requested to confirm the diagnosis. 

**Figure 2:**
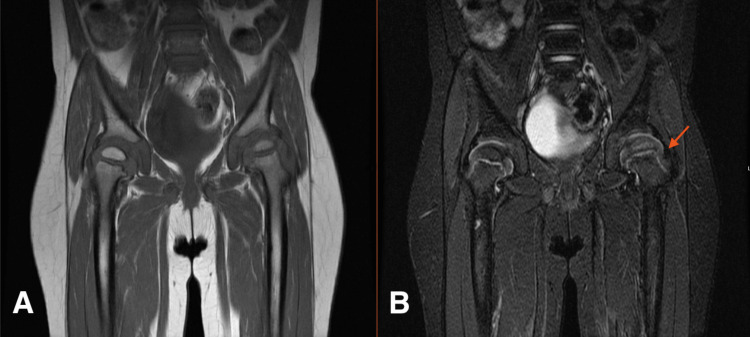
MRI pelvis body T1 (A) and T2 (B). Symmetric bilateral capital femoral and greater trochanter physis signal intensities with no evident SCFE. However, mild diffuse increased T2 signal intensity of the left femoral head (red arrow) with normal height is seen, differentials may include subtle edema for early AVN. SCFE, slipped capital femoral epiphyses; AVN, avascular necrosis

**Figure 3:**
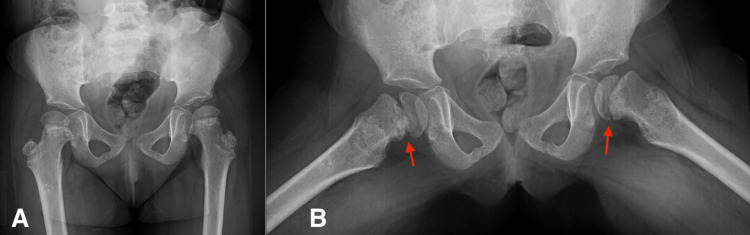
AP pelvis (A) and frog legs lateral (B) X-ray. Redemonstration of bilateral physeal widening, proximal femoral metaphyseal, and iliac crests irregularities, most likely related to chronic kidney disease. On Figure 3B, there is bilateral posterior medial slippage of the femoral epiphysis (red arrows), the right worse than the left, finding are suggestive of bilateral SCFE, with no fractures or gross soft tissue abnormalities. SCFE, slipped capital femoral epiphyses

**Figure 4:**
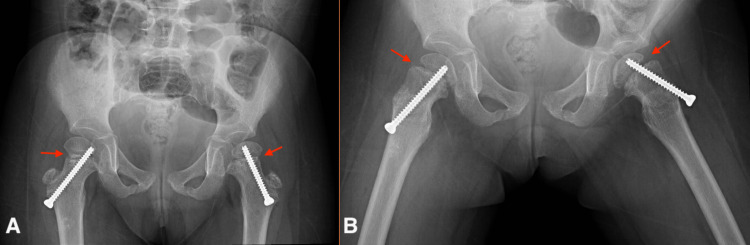
AP pelvis (A) and frog legs lateral (B) X-ray. Status post bilateral in situ pin fixation (red arrows).  No hardware complications.

The MRI (Figure 2A,B) demonstrated no evidence of SCFE, and three musculoskeletal and pediatric radiologists all agreed the MRI was not diagnostic of SCFE based on the known findings in the literature associated with SCFE-like slippage; marrow edema, which appears more on T1 weighted images; physeal winding; and joint effusion, which appears more on T2 weighted images, which was not seen in our patient [8]. Therefore, the radiological changes were thought to be secondary to AVN of her primary disease (skeletal dysplasia). Consequently, a follow-up appointment was scheduled for three months later to conduct another clinical and radiological evaluation of the patient. Upon follow-up, her hip pain was progressing and more severe on the left side. However, her physical examination findings remain unchanged, but the AP pelvis and frog legs lateral X-ray report (Figure 3A,B) showed early slippage of right and left femoral heads. Finally, a diagnosis of bilateral SCFE was made and the patient underwent bilateral in-situ fixation with an uneventful postoperative recovery (Figure 4A,B). 

## Discussion

Slipped capital femoral epiphyses should be considered in pre-adolescents, adolescents, and young adults with atraumatic hip, thigh, or knee pain, especially if associated with limping and inability to bear weight [5]. Whilst SCFE is more common in adolescents, it is also seen in children younger than 10 years old [10]. Chatziravdeli et al. discussed the importance of appropriate diagnosis in children in this age group with clinical manifestations of SCFE [11]. Despite being seven years old, we had a high clinical suspicion of SCFE given our patient’s history of growth hormone treatment and physical examination findings. For this purpose, MRI was ordered to confirm our diagnosis. Upon physical examination, limited internal rotation of the affected hip could be present, and passive internal rotation may elicit pain [5]. Whilst these finding would be easily noted if the contralateral hip were normal, SCFE is commonly bilateral, so careful examination of both hips should be noted [12]. 

Patients with SCFE may demonstrate Drehmann’s sign: obligatory external rotation with passive flexion of the hips to 90 degrees [7]. Weak external foot rotation and hip abduction, decreased hip flexion and internal rotation, and flexion contracture can develop over time [12]. In addition, gait assessment is paramount for diagnosis [13-14]: an out-toeing, abductor lurch, or Trendelenburg gait may suggest SCFE [12]. An abductor lurch gait, described as trunk and hip extension with a compensatory straight knee, is more common than a Trendelenburg gait in SCFE patients [12]. A Trendelenburg gait, dropping off the opposite hip during the stance phase of walking, may suggest gluteus medius weakness or palsy of the gluteal nerve [12]. An out-toeing, is defined as rotational variation of the lower extremity where the feet or toes point away from the midline during gait [12]. In cases with varus or valgus deformity at the knees, patients may compensate by creating a wider stance to maintain normal balance [13-14]. Whilst these signs may be useful, radiology is the proven standard modality of diagnosing SCFE [6]. Radiographs of the contralateral side should always be included to rule out bilateral SCFE [15]. Whilst MRI is more sensitive than conventional radiography in diagnosing SCFE [15], it was inconclusive in our case, and diagnosis was mainly secondary to the clinical findings. 

Physical examination is important in diagnosing SCFE [9]; vague hip, groin, thigh, or knee pain in conjunction with a nontraumatic limp are common findings [8]. Hosseinzadeh et al. outlined the importance of clinical judgment in identifying and diagnosing early SCFE [12]. Delays in treatment are associated with poor clinical outcomes, particularly if the diagnosis has been made eight weeks or more after the initial presentation [12]. Delay in the diagnosis of SCFE is influenced by a number of factors, including family and patient reluctance in seeking medical attention and physician-related delays [16]. Unfortunately, the majority of delayed SCFE diagnoses are due to missed diagnosis [17]; this may lead to multiple complications, including AVN, chondrolysis and femoroacetabular impingement [16]. 

Plain-film radiography, typically AP pelvis and frog leg lateral views of both hips, is the gold standard for confirming an SCFE diagnosis; both are essential views to measure epiphyseal-diaphyseal angle of SCFE [6]. The radiographic signs of SCFE include: widening and irregularities of the physis; relative loss of height of the epiphysis on AP projections; loss of the anterior concavity of the femoral neck on lateral views; the ‘metaphyseal blanch sign’ (a crescent-shaped area of increased density at the proximal and medial femoral neck due to projection of the posterior femoral head, which is displaced posteriorly, inferiorly and medially in relation to the metaphysis); cystic changes at the metaphysis, remodeling, and periost reactions in chronic SCFE; chondrolysis with simultaneous femoral and acetabular subchondral bone changes [15].

The MRI is useful in early stages (pre-slip stage) even when radiographs and CT are normal [18]. However, MRI is considered secondary to conventional radiography in diagnosing SCFE [15, 18]. Early MRI findings include slippage; marrow edema, which appears more on T1 weighted images; physeal winding; and joint effusion, which appears more on T2 weighted images [18]. Previous studies have outlined the importance of MRI in diagnosing SCFE, especially in the early stages [18]. MRI depicts marrow changes earlier than any other imaging method, can assess the risk of chondrolysis and AVN, and has a role in cases where diagnosis is difficult [19]. MRI can identify concurrent, contralateral pre-slips with high sensitivity and specificity prior to surgical treatment, preventing unnecessary prophylactic pinning and indicate surgery for those hips destined to fail [20]. 

## Conclusions

Early diagnosis of SCFE is essential, as a delay in diagnosis will lead to further slippage and increase the risk of additional complications which may eventually irreversibly damage the joint. Although, radiological imaging is the mainstay in diagnosing SCFE especially in its early course, this was not observed in this case. Hence, with proper physical examination and a high degree of clinical judgment, it is recommended that orthopedic surgeons use their clinical sense and examination skills to diagnose SCFE promptly, in order to maintain a short follow-up window to prevent any delay in surgical management and to observe for any progression, even if the radiological findings are normal.
